# Glassy Powder Derived from Waste Printed Circuit Boards for Methylene Blue Adsorption

**DOI:** 10.3390/molecules29020400

**Published:** 2024-01-13

**Authors:** Saad Javaid, Alessandra Zanoletti, Angela Serpe, Elza Bontempi, Ivano Alessandri, Irene Vassalini

**Affiliations:** 1 Sustainable Chemistry and Materials Laboratory, Department of Information Engineering, University of Brescia, Via Branze 38, 25123 Brescia, Italy; s.javaid@studenti.unibs.it (S.J.); ivano.alessandri@unibs.it (I.A.); 2 Chemistry for Technologies Laboratory, Department of Mechanical and Industrial Engineering, University of Brescia, Via Branze 38, 25123 Brescia, Italy; alessandra.zanoletti@unibs.it; 3 Unit of National Interuniversity Consortium for Materials Science and Technology (INSTM), Research Unit of Brescia, Via Branze 38, 25123 Brescia, Italy; 4 Department of Civil and Environmental Engineering and Architecture (DICAAR), INSTM Unit, Via Marengo 2, 09123 Cagliari, Italy; serpe@unica.it; 5 National Research Council of Italy, Institute of Environmental Geology and Geoengineering (CNR-IGAG), Via Marengo 2, 09123 Cagliari, Italy; 6 CNR-INO (National Research Council-National Institute of Optics), Research Unit of Brescia, Via Branze 38, 25123 Brescia, Italy

**Keywords:** methylene blue, adsorption, e-waste, recycling, printed circuit boards, glass fibers, water remediation

## Abstract

Electronic waste (e-waste) is one of the fastest-growing waste streams in the world and Europe is classified as the first producer in terms of per capita amount. To reduce the environmental impact of e-waste, it is important to recycle it. This work shows the possibility of reusing glassy substrates, derived from the MW-assisted acidic leaching of Waste Printed Circuit Boards (WPCBs), as an adsorbent material. The results revealed an excellent adsorption capability against methylene blue (MB; aqueous solutions in the concentration range 10^−5^ M–2 × 10^−5^ M, at pH = 7.5). Comparisons were performed with reference samples such as activated carbons (ACs), the adsorbent mostly used at the industrial level; untreated PCB samples; and ground glass slides. The obtained results show that MW-treated WPCB powder outperformed both ground glass and ground untreated PCBs in MB adsorption, almost matching AC adsorption. The use of this new adsorbent obtained through the valorization of e-waste offers advantages not only in terms of cost but also in terms of environmental sustainability.

## 1. Introduction

Over the past few years, there has been a dramatic surge in electronic waste (e-waste) [1], primarily driven by the widespread consumption of electronic devices, and also due to the increased home activities, such as smart working, during COVID-19 [2].

**Figure 1:**
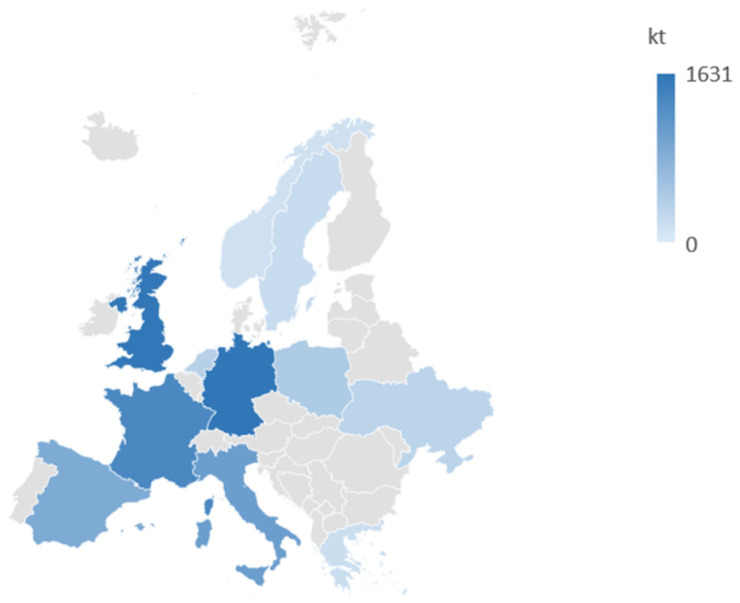
E-waste production in some European countries. Data taken from ref. [3].

In 2019, Norway led the world in per capita e-waste generation, with 28.5 kg per person, just slightly ahead of the United Kingdom and Denmark. During that year, the global e-waste generation surged to around 54 million tons (worldwide; 7.3 kg per capita), with 12 million tons produced in Europe and more than 1 million in Italy alone. Data are summarized in Figure 1 [3].

Moreover, the substantial annual waste generated is expected to continuously increase in the coming years, reaching a value of ~9 kg per capita in 2030, with a global production of about 75 Mt. Consequently, manufacturers have intensified their efforts in the electronics recycling sector, focusing on the production of reconditioned and recycled electronic devices. From 2022 to 2030, a substantial increase in the electronics recycling market is expected. If in 2022, this market’s size stood at approximately 40 billion U.S. dollars, it is expected to attain a value of 110.6 billion U.S. dollars by 2030, with an annual growth rate of 13.6% [4].

Memory cards, such as SD cards, microSD cards, or compact flash cards are examples of e-waste of increasing interest in the recycling market. Unfortunately, they typically contain a combination of materials and units, making their recycling very complex and expensive. So, even if recycling e-waste and reusing their components, with a similar purpose to the original one, remain the preferred approaches, the recovery of all the components is often hindered by the intricate nature of electronic devices [5]. Extended activities in research and procedure optimization have been performed in this sense, leading to the development of specific recycling chains, after the preliminary steps of dismantling, sorting, and mechanical pretreatment (such as shredding and crushing) [6,7,8].

For example, memory cards, as well as other electronic waste, are often housed in plastic casings, which can be recycled and used in the production of new plastic products [9,10]. Similarly, any paper or plastic labels and printing on the memory card’s surface can be separated, and the materials may be recycled or disposed of properly. Simultaneously, memory cards have various metal contact pins or connectors made of precious materials like gold or silver, which can be extracted and refined for reuse or resale [11,12]. Furthermore, memory cards have a small Printed Circuit Board (PCB) responsible for electrical interconnections among different elements. Also, PCBs have an intricate composition: for ~40% wt, they are composed of metals (mainly Cu, Sn, Fe, and Pb, with fractions of Au, Ag, and Pd used as contact materials or plating layers thanks to their electric conductivity and chemical stability); for ~30% wt, they are composed of polymers; while the remaining ~30% wt is made of ceramic–polymeric materials. For ~23% wt, this last component is made of a glassy plastic support, called fiberglass, composed of glass fibers included in an epoxy resin matrix [13]. As anticipated, up to now, the main focus of the recovery processes has been on precious metals that can be extracted from e-waste and recycled [14,15,16,17], but waste PCBs (WPCBs) can be repurposed more extensively as a reservoir of raw materials for diverse applications, following a circular economy approach. Considering that any electric and electronic device contains at least one PCB, and that PCBs represent ~8% of the weight of small electronic devices, their recycling is fundamental to reduce the amount of electronic waste. At the same time, they can be considered real “urban mining” sources, enabling the recovery of valuable resources and contributing to sustainability efforts [18]. For example, elastomeric components, such as rubbers or polyurethanes, can exhibit shape memory properties and can be reused in various applications [19], or epoxy resin and polymeric materials may be incinerated for energy recovery [20,21].

The packaging material usually also contains a high amount of silica, which can be easily converted into mesoporous silica and subsequently used as a template for the synthesis of mesoporous carbon [22]. The polymeric components are mainly thermoset resins and reinforcement materials, which can barely be recycled because their crosslinked structure makes their melting impossible using conventional processes. Traditionally, these materials are landfilled or incinerated, but recently their recovery and use as fillers for epoxy or polypropylene resin products, such as paints, adhesives, decorating agents, and building materials, have been proposed [8,23,24].

However, despite the continuous increase in the number of research activities devoted to recycling e-waste, there are only sporadic works focused on the recovery and reuse of glass fibers derived from WPCBs after the extraction of the most valuable components [25], especially in relation to their conversion into adsorbent materials. Furthermore, their field of application is limited to the removal of inorganic ions from water [26,27,28,29,30].

In this work, we aimed to investigate the possibility of valorizing fiberglass residual, converting it into an easily usable adsorbent for the removal of methylene blue (MB) from water. MB is a synthetic dye which is widely used in a large variety of sectors: it is used as a colorant for papers, wool, silk, and cotton, but it is also employed in the food, cosmetics, and pharmaceuticals industries. Although it can be used for some medical treatments, uncontrolled intake via contaminated water can expose humans to various ailments. Similarly, high concentrations of MB in water can create serious hazards to the whole ecosystem [31]. Unfortunately, MB has a strong affinity for water, and it (bio)degrades with difficultly, leading to its accumulation, especially in Asian countries, where the number of textile industries pouring contaminated effluents in natural watercourses is high. The same considerations are valid for various synthetic organic dyes. Their removal through conventional wastewater treatment is limited, and this fact has led the scientific community to devise innovative methods, such as electrocoagulation, photodegradation, biodegradation, or adsorption [32]. Among them, adsorption, consisting of the attraction and anchoring of solute molecules onto the surface of a solid, is one of the favorite strategies thanks to simplicity and affordability, and the high availability of low-cost adsorbent materials [33,34,35], frequently deriving from biomass or other waste materials [36,37]. In this context, developing high-added-value adsorbent materials from the so-far unexploited residuals of e-waste is an exciting area of study which enables researchers to find a common answer to a couple of environmental concerns: water pollution and waste disposal.

## 2. Results and Discussion

**Figure 2:**
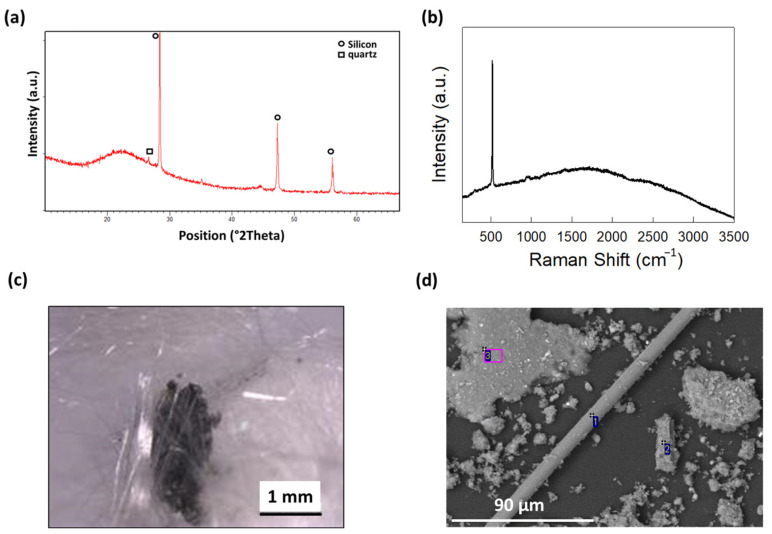
Characterization of MW-treated WPCB samples: (**a**) X-ray diffraction pattern; (**b**) Raman spectrum; (**c**) Stereoscopic Optical Microscopy; (**d**) Scanning Electron Microscopy.

Waste Printed Circuit Boards (WPCBs) were collected and treated through acidic leaching assisted by microwave heating, as reported in the Materials and Methods section (Section 3), to obtain a solid fraction (MW-treated WPCBs) depleted of metal components and epoxydic resin. The obtained material was analyzed through X-ray diffraction and Raman Spectroscopy to obtain information about its specific chemical composition. As visible from Figure 2a, it was mainly composed of an amorphous fraction, ascribed to amorphous SiO_2_ (glass), and crystalline Si, whose diffraction peaks were visible at 2θ = 28.4°, 47.3°, and 56.1°. A small contribution to the diffraction pattern came also from quartz (crystallin SiO_2_), with a small peak centered at 2θ = 26.6°.

In Figure 2b, the Raman spectrum is reported, confirming the presence of crystalline Si (Raman peak centered ad 520 cm^−1^), while no impurities deriving from additional original components of PCBs were detected. These data demonstrated that the grinding and MW-assisted acidic digestion with HNO_3_, HCl, and H_2_O_2_ enabled the removal of the epoxy resin originally contained in the fiberglass substrate (together with glass fibers) and almost all the metallic components (Au, Cu, Ag, Ni, Fe, Al, Mn, Pb, Pb, Sn, Cr, and Zn) previously contained in the WPCBs, in accordance to that reported in [16]. The permanence of crystalline Si, instead, was due to the fact that HF was not used during the leaching process. The obtained powder was also investigated through optical and electronic microscopy, which enabled us to confirm the presence of Si residues and proved the presence of the morphology typical of glass fibers (Figure 2c,d). In Section S1 of the Supplementary Materials, the corresponding energy-dispersive X-ray spectroscopy analysis is reported, which elucidated the presence of minor metallic impurities corresponding to Al, Ti, Cr, and Fe, together with C, F, Ca, and Cl.

**Figure 3:**
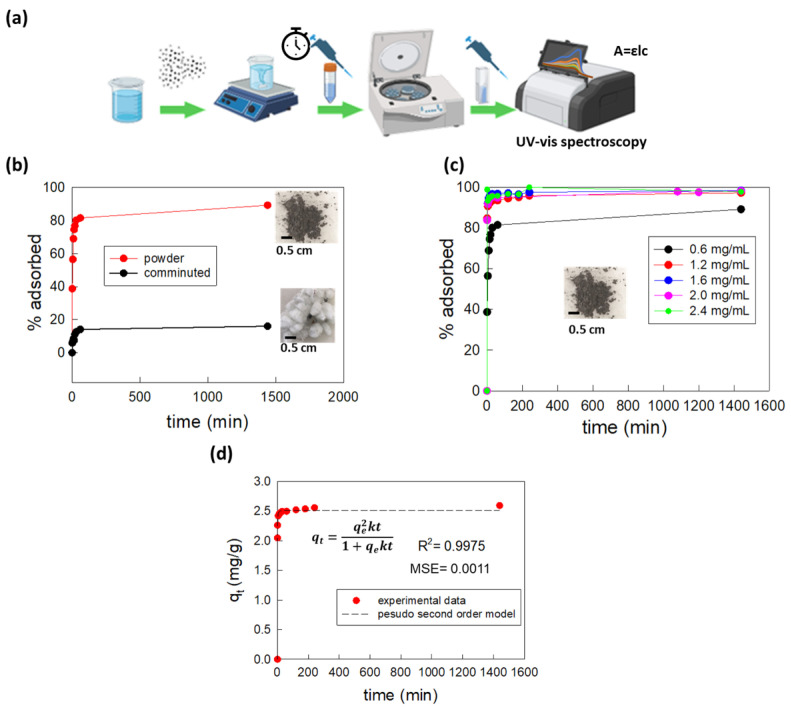
Investigation of the adsorption process of MB (10^−5^ M) on MW-treated WPCB samples at pH = 7.5: (**a**) schematic representation of the adsorption experiments, in accordance with what is described in the Materials and Methods section (Section 3); (**b**) comparison between the percentages of adsorbed MB 10^−5^ M in the time interval 0–24 h, using as the adsorbent MW-treated WPCB samples with different granulometry: comminuted fragments of 0.3 cm^2^ (blackline) or fine powder with granulometry <500 μm (red line); (**c**) comparison between the percentages of adsorbed MB 10^−5^ M in the time interval 0–24 h using different amounts of powder derived from MW-treated WPCBs; (**d**) variation in the adsorption capacity of the powder derived from MW-treated WPCBs at the dosage of 1.2 mg/L as a function of soaking time inside the MB 10^−5^ M solution. Points represent the experimental data, while the dashed line represents the results of the fitting using the non-linearized form of the pseudo-second-order model. The high value of the correlation coefficient (R^2^) and the low value of the mean sum of squares error (MSE) indicate the goodness of the fit. All the experiments were conducted in triplicate; error bars are included inside dots’ size. Figure 3a was created with BioRender.com, accessed on 10 January 2024.

The MW-treated WPCB samples were tested as adsorbents for the removal of organic dyes from mineral water (see Table S1 for water chemical analysis) by putting known amounts of solid samples in contact with 5 mL of methylene blue (MB) 10^−5^ M solutions (measured pH = 7.5) and evaluating the variation in the absorption spectra of the dye solution over the time, as schematized in Figure 3a. The procedure for the adsorption process was kept simple on purpose (no pH variation or buffering, simple agitation by means of magnetic stirring, no heating) in order to match conditions easily applicable in the real world.

Initially, the effect of the granulometry of the MW-treated WPCB samples on the adsorption efficiency was investigated by comparing the adsorption percentage of MB achieved using 3 mg of comminuted MW-treated WPCBs or 3 mg of pulverized MW-treated WPCBs (dosage of adsorbent = 0.6 mg/mL). The results reported in Figure 3b clearly show that further grinding and pulverization after MW acidic treatment of WPCBs was a fundamental step to achieve high adsorption percentages. In fact, in the case of macroscopic fragments (size~0.3 cm^2^), the removal of MB, even after 24 h of contact and stirring, was very low (limited to 16%), while it significantly increased up to 89% after proper grinding through a ball mill, obtaining a powder with a granulometry lower than 500 μm. The enhancement of adsorption was evident immediately, just after the first intervals of contact: the pulverized sample was able to outperform the adsorption percentage achieved in 24 h using the coarse-grained samples in less than 1 min (removal efficiency of 38.7%). The reason for this behavior is linked to the fact that smaller granulometry entailed a higher specific surface area, which is an ideal condition for adsorption processes. For this reason, all the subsequent adsorption tests were performed on pulverized MW-treated WPCB samples.

Adsorption could also be enhanced by increasing the amount of adsorbent material, as illustrated in Figure 3c, where the % of removed MB achieved by adding 3, 6, 8, 10, or 12 mg in 5 mL of solution are compared. The most significant difference was observed when passing from a dosage of adsorbent material of 0.6 mg/mL (3 mg in 5 mL of MB solution) to 1.2 mg/mL (6 mg in 5 mL of MB solution), while further dosage increments led to almost un-noticeable adsorption variations: small variations were limited to the first minutes of the adsorption process, while the equilibrium value reached after 24 h remained constant (MB removal efficiency ~98%). For these reasons, all further experiments were conducted considering the adsorbent dose of 1.2 mg/mL.

The obtained data were used for the calculation of the adsorption capacity of the powder derived from MW-treated WPCBs, q_t_, corresponding to the amount (mg) of MB adsorbed on their surface over time and normalizing it towards the amount of adsorbent material (g). The variation in q_t_ as a function of the contact time between the powder and the dye solution is reported in Figure 3d, showing that most of MB was adsorbed in the first 5 min, after which q_t_ tended to plateau, thanks to an equilibrium being reached between the adsorption of MB molecules on the powder surface and their desorption into the surrounding solution. These features are typical of adsorption processes characterized by a kinetic of the pseudo-second order. Further details regarding the fitting of the adsorption data of MW-treated WPCB powder according to different kinetic models are reported in Section S3 of the Supplementary Materials.

From these results, it was evident that the powder derived from MW-treated WPCBs enabled almost complete MB removal in very easy experimental conditions: no pH buffering, no addition of chemicals, room temperature, and simple agitation. We tried to extend the application of these adsorbents to a higher MB concentration, doubling their value to 2 × 10^−5^ M (Figure S3). Also in this case, very satisfactory results were obtained: after 1 min of contact and stirring, the powder enabled the removal of 77.5% of the initial MB (in the case of MB 10^−5^ M, the % removed in the first minute was 84.8%), which rapidly increased up to 89.9% at 5 min and reached the equilibrium value of 98.9% after 24 h, even surpassing the value obtained in the case of MB 10^−5^ M solution). Again, the adsorption process followed a kinetic of the pseudo-second order. Interestingly, the obtained value for the equilibrium adsorption capacity (5.27 ± 0.007 mg/g) was more than double the adsorption capacity obtained in the case of MB 10^−5^ M solution (2 × 2.59 = 5.18 mg/g), suggesting that the MW-treated WPCB powder could adsorb an even higher amount of MB.

The adsorption performances of the MW-treated WPCB powder against MB 10^−5^ M were compared with reference samples: commercial activated carbons (ACs), untreated PCBs, and glass. All the samples were considered in the form of powder (untreated PCBs and glass were ground, as described in the Materials and Methods section (Section 3)), and all the experimental conditions (MB concentration, not-buffered pH (pH = 7.5), mineral water, room temperature, stirring at 700 rpm, adsorbent dosage of 1.2 mg/mL) were maintained constant.

Activated carbons were selected because they can be considered the commercial standard for industrial adsorption processes; untreated PCBs were selected to verify if the performed MW treatment and acidic leaching influenced the adsorption process; glass was selected as reference material with a chemical composition similar to that of MW-treated WPCB samples.

**Figure 4:**
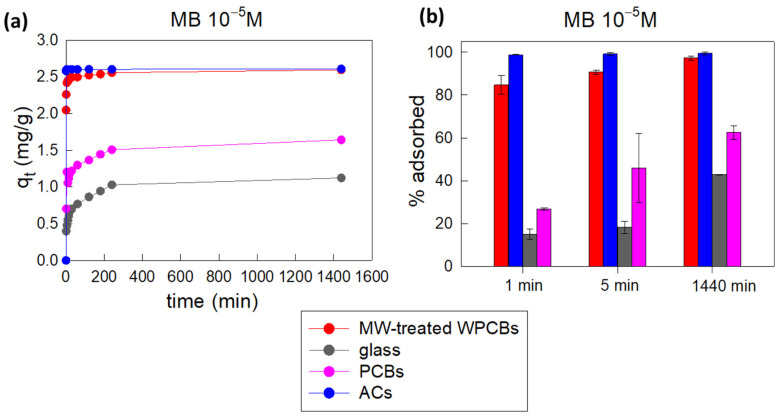
Comparison between the adsorption capacity of MW-treated WPCB powder and those of reference materials (ACs, glass powder, and ground untreated PCBs) during the adsorption of MB (10^−5^ M): (**a**) variation in adsorption capacities as a function of time in relation to MB 10^−5^ M; (**b**) comparison between the percentage of adsorbed MB 10^−5^ M at selected time intervals. All the experiments were conducted in triplicate, where non-visible error bars are included inside dots’ size.

The obtained results in terms of q_t_ are reported in Figure 4a, while the correlated percentage of adsorbed MB 10^−5^ M for selected significant time intervals is reported in Figure 4b.

From these data, it is evident that the MW-treated WPCB powder outperformed both ground glass and ground untreated PCBs in MB adsorption, almost matching ACs. It seems that Si and other minor impurities contaminating the glass matrix in the MW-treated WPCBs sample, as well as the fiber morphology, enabled a better interaction with MB in comparison to simple glass. In particular, the morphological effect has already been observed by Chakrabarti and Dutta [38], who showed that glass fibers contain microcracks capable of harboring MB molecules, leading to higher adsorption in comparison to borosilicate glass.

Data reported in Figure 4a,b also suggest that metal leaching through MW acidic treatment was fundamental to maximize MB adsorption, probably thanks to an increase in the material porosity and surface area, as well as a reduction in positive charges on the adsorbent surface due to metals. During this process, various metals were removed from the fiberglass substrate, leaving empty spaces and enabling more direct contact between the dye molecules in the solution and silica, which is the primary constituent of glass. Considering that at pH = 7.5 MB is a cationic dye and silica is negatively charged at pH > 3 (pzc of SiO_2_ 2–4 [39]), the above-described conditions are ideal for enhancing MB adsorption. However, it was quite surprising that the powder derived from the MW-treated WPCBs was able to almost match the adsorption performances of ACs: even if ACs were characterized by a slightly faster adsorption process in the first minutes, the two types of materials reached the same equilibrium values of q_t_ (2.59 ± 0.02 mg/g for MW-treated WPCBs and 2.61 ± 0.02 mg/g for ACs) and percentages of adsorbed MB (97.3 ± 0.9% for MW-treated WPCBs and 99 ± 1% for ACs) in 24 h. Given that the MW-treated WPCB powder was obtained from a waste material, it can be considered a very promising alternative to commercial adsorbents, characterized by lower price and higher environmental sustainability. Additionally, ACs and MW-treated PCBs seemed to be characterized by the same kinetic behavior, since their experimental data can be satisfactorily fitted following a pseudo-second-order kinetic model (see Figure S4a): very fast adsorption occurred in the first few minutes, followed by a prolonged process of continuous adsorption/desorption of MB molecules, leading to a dynamic equilibrium being reached. In the case of glass and untreated PCBs, adsorption instead occurred more gradually and the best kinetic model for the fitting of the experimental data resulted to be the Elovich model (Figure S4b,c).

In view of these promising results, we investigated the possibility of using MW-treated WPCB powder for the adsorption of methylene orange (MO), and we compared its activity with that of ACs, glass powder, and not-treated PCB powder. The same experimental conditions were considered: adsorbent materials at the dosage of 1.2 mg/mL, room temperature, no pH buffering (pH = 7.5), dye concentration equal to 10^−5^ M, and mineral water. The obtained results are reported in Figure S5a (variation in q_t_ as a function of time) and S5b (comparison between the percentage of MO adsorbed at selected time intervals). It is clearly visible that in this case, the MW-treated WPCB powder was able to remove only a limited percentage of the dye and had a significantly lower adsorption capacity in comparison to commercial ACs.

While MB is a cationic dye, MO is characterized by a negative charge at approximately neutral pH (experimental pH of dye solutions was 7.5). One of the reasons for the different behavior of the MW-treated WPCB powder could be linked to the fact that it adsorbed dye mainly through electrostatic interactions, thanks to the presence of a net electrostatic charge on its surface. We measured the surface charge of ground MW-treated WPCBs through Dynamic Light Scattering, and it resulted to be slightly negative, equal to −23 ± 6 mV, in line with the value reported by Tsai and Horng [40] in relation to waste fiberglass. Consequently, they should be capable of adsorbing cationic dyes, like MB, while they would not be ideal for the interaction and removal of anionic dyes, such as MO. We also measured the surface charge of Acs, which resulted to be negative too (−26 ± 1 mV). However, ACs are composed of piled stacks of 2–10 small aromatic and graphitic layers with a size of about 1 nm combined with disorganized carbon units, mainly aliphatic chains, which are present in the periphery of the aromatic layers and work as cross-linkage structures. This “turbostratic structure” was confirmed in the batch of ACs we used as reference, as demonstrated by the XRD pattern reported in Figure S6a, and was characterized by the presence of two broad peaks centered at 25°, corresponding to the (002) set of planes, and at around 44°, which corresponds to the (100/101) set of planes [41]. Each of the piled stacks was mainly characterized by a graphitic structure, as visible in the associated Raman spectrum reported in Figure S6b, which shows only the peaks ascribable to the typical D (associated with sp^3^ carbon atoms and defects in a graphene layer) and G bands (associated with the sp^2^ carbon atoms forming the graphene skeleton), centered at 1350 cm^−1^ and 1600 cm^−1^, respectively, with no contribution from other organic functional groups, in accordance with previous studies [42].

The above-described organic structure with a high number of aromatic units enables the interaction with and adsorption of various organic dyes and pollutants, independently from their charge, thanks to the insurrection of π-π interactions. This fact, together with the well-known enhanced specific surface area, can justify the higher performance of ACs, as regards both MB and MO.

From Figure S5, it is visible that both glass powder and the powder obtained from untreated PCBs were able to absorb a slightly higher percentage of MO in comparison to MW-treated WPCBs. Even if their adsorption capabilities were significantly lower than that of ACs, after 24 h glass removed ~12% of MO, and untreated PCBs removed ~23% of MO, leading to adsorption efficiencies higher than the ~5% achieved by the MW-treated WPCB samples. As reported in Figure S7a, the X-ray diffractogram of untreated PCBs was characterized by the presence of peaks representative of crystallin copper, aluminum silicate, and FeAl_2_, in addition to the features typical of quartz and amorphous silica, also maintained in the samples which underwent the MW treatment. The presence of these components at the atomic level at a higher concentration in comparison to the MW-treated WPCBs was also confirmed through SEM and EDX analysis (Figure S7c). Furthermore, the MW treatment enabled the removal of the epoxy resin, which, together with glass fibers, constituted the fiberglass substrate. Its presence, instead, was clearly visible in the Raman spectrum reported in Figure S7b, which was characterized by the presence of peaks centered at 690 cm^−1^ (aromatic C-H out-of-plane deformation), 770 cm^−1^ (C3-C2 skeletal), 1220 and 1290 cm^−1^ (C-O stretching, 1450 cm^−1^ due to -CH_3_ bending), and 1550 cm^−1^ (stretching of C=C bonds of the aromatic rings) [43].

It is probable that this organic component was responsible for the increased MO adsorption in the case of untreated PCB samples. Similarly to what happened in the case of ACs, it could improve π-π interactions between the solid and the organic dye in solution, facilitating its adsorption. However, untreated PCBs also did not enable a complete removal of MO, maintaining performance values well under those achieved by employing commercial adsorbents. Further studies will be performed to extend the activity of MW-treated WPCBs to the adsorption of anionic dyes, such as MO, or other classes of water contaminants, and to test the possibility of subsequent degradation of adsorbed pollutants, but these promising results related to MB adsorption pave the way for the development of systems capable of environmental remediation starting from e-waste.

## 3. Materials and Methods

### 3.1. MW-Treated WPCB Samples’ Preparation and Characterization

The MW-treated WPCB samples represent the non-metal fraction deriving from waste Random Access Memory (RAM) electronic boards obtained as a solid residue of their acidic digestion. This fraction counts for the 40% wt of the starting material. Specifically, 200 g of RAMs of different origin were roughly milled for 24 h in a stainless-steel jar by means of hard metal balls (Ø 6 mm, 1.4 kg) in a planetary apparatus (4-stage Retsch mill, 300 rpm) in the presence of Carbsyn 110 (250 mL), as described in [16]. The final slurry was separated from the hard metal balls using sieves with grates of 4 mm. Then, Carbsyn was recovered via distillation, and the milled sample was collected and dried. Aliquots of the comminuted sample were weighed and then digested under microwaves into TFM vessels containing a mixture of HNO_3_ (65%, 2 mL), HCl (37%, 6 mL), and H_2_O_2_ (30%, 0.5 mL). The treatment was performed using a Milestone Ethos 1 Microwave digester (Milestone srl, Sorisole (BG), Italy), equipped with an HPR1000/10S high-pressure segmented rotor, an ATC-400CE automatic temperature control, and a Terminal 640 with easy-CONTROL software, applying a microwave program consisting of two steps lasting 10 and 20 min, respectively, at a temperature of 220 °C and microwave power up to 1000 W. Digested samples were washed three times with Milli-Q water and once with acetone, and left to dry.

The obtained comminuted fraction was characterized by a granulometry of about 0.3 cm^2^, and it was further ground through ball milling (MM400, Retsch, Haan, Germany) until reaching a fine powder: 2–4 mL of samples corresponding to ~25 mg of MW-treated WPCB fragments were inserted in a stainless-steel jar (total volume: 10 mL) together with 7 steel balls (Ø 7 mm) and the instrument was activated at a frequency of 25 Hz for 3 min. The obtained powder was sieved with grates of 500 μm.

The crystallographic structure and chemical composition of the obtained powder were investigated usin X-ray diffraction (Panalytical (Malvern, UK) diffractometer using Cu Ka (1.5406 Å) radiation and operating at 40 kV and 40 mA) and Raman Spectroscopy (Labram HR-800 spectrophotometer (Horiba/Jobin Yvon, Kyoto, Japan) equipped with a He−Ne laser source (λ = 632.8 nm); the acquisition time was 10 s, laser power was attenuated to 0.3 mW (10% filter)). Its morphology was investigated through Stereoscopic Microscopy (Leica MZ 16 A coupled with software Leica Qwin (Leica, Wetzlar, Germany) and Scanning Electron Microscopy (FEI NOVA 600 (FEI, currently Thermofisher, Eindhoven, The Netherlands)).

The surface charge was measured through Dynamic Light Scattering (DLS, Nanolink S900, Linkoptik (Zhuhai, China)).

### 3.2. Reference Adsorbents Preparation and Characterization

The adsorption performances of the MW-treated WPCB powder were compared with those of commercial activated carbon (DARCO, Sigma Aldrich, St. Louis, MO, USA) and powders obtained from not-treated PCBs and glass slides. In particular, a glass slide and a PCB sample were manually fragmented and subjected to ball mill fragmentation: ~2 mL of each sample (~100 mg) was inserted in a stainless-steel jar (total volume: 10 mL) together with 7 steel balls (Ø 7 mm), and the instrument was activated at a frequency of 25 Hz. The grinding process lasted for 3 min in the case of glass, while it was extended up to 9 min in the case of PCB samples. Both the obtained powders were sieved with grates of 500 μm. Their characterization was performed similarly to what is reported in the previous section in relation to the MW-treated WPCBs.

### 3.3. Adsorption Experiments

Known amounts (3, 6, 8, 10, 12 mg) of MW-treated WPCB samples were added to 5 mL of methylene blue (MB) or methyl orange (MO) solutions with a concentration of 10^−5^ M prepared using mineral water (chemical analysis reported in Table S1), and they were left under stirring at 700 rpm for at least 24 h. In the case of MB, concentration 2 × 10^−5^ M was also tested. After regular time intervals (1, 5, 10, 15, 20, 30, 60, 120, 180, 240, and 1440 min), each sample was centrifuged at 9000 rpm for 4 min at 22 °C (Neya 16R centrifuge) to separate the solid from the liquid phase, which was analyzed through UV-vis spectroscopy (QE 65000 Ocean Optics Spectrometer, Orlando, FL, USA).

Absorption spectra were recorded and the changes in the absorbance intensity at 660 nm for MB and at 460 nm for MO were measured.

The percentage of adsorbed pollutant was calculated as follows:

$$\%\;Adsorbed=\frac{A_{0}-A_{t}}{A_{0}}\times 100$$

where A_0_ corresponds to value of absorbance of the starting solution of MB or MO and A_t_ corresponds to the absorbance measured at time t, after the addition of the adsorbent material. The obtained data were used for the calculation of q_t_, which represents the amount of adsorbed MB or MO per gram of adsorbent (mg g^−1^) at any time t (min). At the end of the adsorption test, it was possible to calculate the value of q_e_ (mg g^−1^), which corresponds to the equilibrium adsorption capacity.

The experimental data were fitted using the Solver add-in program of Excel using the not-linearized equations of the pseudo-first-order model, pseudo-second-order model, Elovich model, and intraparticle diffusion model [44]. Further details can be found in Section S3 of the Supplementary Materials.

The adsorption performances of the powder derived from MW-treated WPCBs were compared with the adsorption capacity of analogous powders obtained from not-treated PCBs, glass slides, and commercial activated carbon (DARCO), keeping all the experimental parameters constant (room temperature, sampling times, mineral water, 10^−5^ M dye concentration, no pH adjustment, adsorbent dose of 1.2 mg/mL).

All the experiments were performed in triplicate.

The pH of the solutions was measured by means of the SI Analytics, Lab 845, pH meter and resulted to be 7.5.

## 4. Conclusions

In this work, we demonstrated that by performing acidic digestion assisted by MW heating it was possible to leach metals and epoxy resin from waste PCBs, leading to the production of a Si-enriched glassy substrate. After its grinding into a powder with a granulometry lower than 500 μm, it was possible to achieve a powder characterized by an excellent adsorption capability against MB (in the concentration range 10^−5^ M–2 × 10^−5^ M, pH = 7.5). Although its adsorption performance was limited to cationic dye, it was able to match the activity of commercial activated carbons during MB removal, significantly outperforming similar powders obtained from the grinding of glass slides or un-treated PCB samples. The here-developed adsorbent was characterized by an intrinsic low cost, since it was obtained through the valorization of wastes, limiting not only the costs of raw materials but also those of the disposal of the original waste, with clear advantages in comparison to commercial activated carbons (~250 EUR/kg). Furthermore, following this approach based on the principles of circular economy, it was possible to limit the CO_2_ emissions linked to the conventional disposal of PCBs in landfills, paving the way for the development of highly sustainable adsorbents and extending the potential sectors in which e-waste can find application through appropriate recycling processes.

## Data Availability

The data are reported within the article and in the Supplementary Materials.

## Supplementary Materials

The following supporting information can be downloaded at https://www.mdpi.com/article/10.3390/molecules29020400/s1, Section S1: EDX analysis of MW-treated WPCBs; Section S2: Chemical analysis of mineral water used for the preparation of dye solutions; Section S3: Identification of the best kinetic model for the fitting of MB adsorption data of MW-treated PCBs; Section S4: Adsorption of MB at various concentrations; Section S5: Fitting of MB adsorption data obtained using different adsorbent materials; Section S6: Adsorption of MO 10^−5^ M; Section S7: Structural and chemical characterization of activated carbons, used as reference material; Section S8: Structural and chemical characterization of powder derived from untreated PCBs, used as reference material.
