# Supplementary material for: Glassy Powder Derived from Waste Printed Circuit Boards for Methylene Blue Adsorption
Source: Molecules. 2024 Jan 13;29(2):400. doi: 10.3390/molecules29020400 (PMC10821274; doi:10.3390/molecules29020400)
Supplement: Supplementary file 1 [file molecules-29-00400-s001.zip › molecules-2805799-supplementary.pdf]

Glassy Powder Derived from Waste Printed Circuit Boards for Methylene Blue Adsorption

Saad Javaid [1], Alessandra Zanoletti [2,3], Angela Serpe [4,5], Elza Bontempi [2,3,*], Ivano Alessandri [1,3,6] and Irene Vassalini [1,3,6,*]

[1] Sustainable Chemistry and Materials Laboratory, Department of Information Engineering, University of Brescia, Via Branze 38, 25123 Brescia, Italy; s.javaid@studenti.unibs.it (S.J.); ivano.alessandri@unibs.it (I.A.)
[2] Chemistry for Technologies Laboratory, Department of Mechanical and Industrial Engineering, University of Brescia, Via Branze 38, 25123 Brescia, Italy; alessandra.zanoletti@unibs.it
[3] Unit of National Interuniversity Consortium for Materials Science and Technology (INSTM), Research Unit of Brescia, Via Branze 38, 25123 Brescia, Italy
[4] Department of Civil and Environmental Engineering and Architecture (DICAAR) and INSTM Unit, Via Marengo 2, 09123 Cagliari, Italy; serpe@unica.it
[5] National Research Council of Italy, Institute of Environmental Geology and Geoengineering (CNR-IGAG), Via Marengo 2, 09123 Cagliari, Italy
[6] CNR-INO (National Research Council-National Institute of Optics), Research Unit of Brescia, Via Branze 38, 25123 Brescia, Italy
* Correspondence: elza.bontempi@unibs.it (E.B.); irene.vassalini@unibs.it (I.V.)

Section S1. EDX analysis of MW-treated WPCBs

Section S2. Chemical Analysis of mineral water used for the preparation of dyes solutions

Section S3. Individuation of the best kinetic model for the fitting of adsorption data

Section S4. Adsorption of MB at various concentrations

Section S5. Pseudo-second order model fitting of MB adsorption considering different adsorbent materials

Section S6. Adsorption of MO 10^{-5} M

Section S7. Structural and chemical characterization of activated carbons, used as reference material.

Section S8. Structural and chemical characterization of powder derived from untreated PCBs, used as reference material.

Section S1. EDX analysis of MW-treated WPCBs

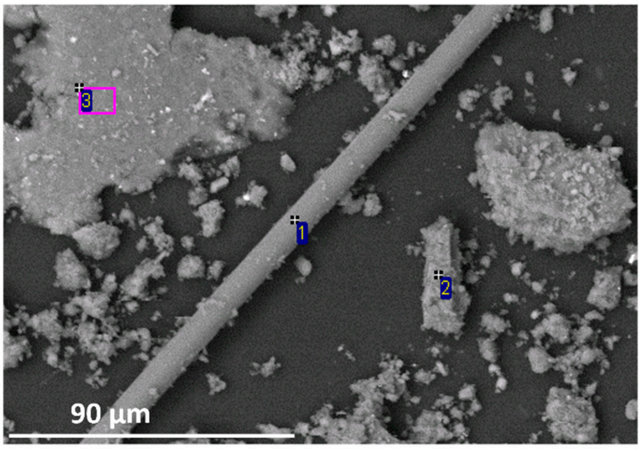

	Spectrum	C	O	F	Al	Si	Cl	Ca	Ti	Cr	Fe	Cu
						wt (%)						
	1		71.44		1.53	24.69		2.33				
a	2	29.38	55.42		0.27	12.69	0.35	0.16			1.72	
	3	27.77	47.14	4.34	0.34	16.38	0.51	0.42	0.31	0.51	2.29	

Figure S1. SEM image and corresponding EDX analysis of ground MW-treated WPCB

Section S2. Chemical Analysis of mineral water used for the preparation of dyes solutions

Substances dissolved in one litre of water expressed in ions			
Ca^{2+}	11.2 mg/L	SO_4^{2-}	5.6 mg/L
Mg^{2+}	3.5 mg/L	NO_3^-	3.8 mg/L
Na^+	2.0 mg/L	Cl^-	2.0 mg/L
K^+	0.70 mg/L	F^-	< 0.1 mg/L
HCO_3^-	50 mg/L	SiO_2	7.1 mg/L

Table S1. Chemical composition of bottled water. Chemical-physical constants: Water temperature at the source: 10.8°C · pH at the source: 7.60 · specific electrical conductivity at 20°C: 91 µS/cm · fixed residue at 180°C. 60 mg/L· Hardness °f 5.9 · free CO_2 at the source: 6.0 mg/L.

Section S3. Individuation of the best kinetic model for the fitting of MB adsorption data of MW-treated PCBs

From the experimental adsorption data and through the following formula:

$$q_t = (C_0 - C_t) \times (V/m)$$

where C_0 represents the starting concentration of the dye (mg/L), C_t represents the concentration of the dye at a particular sampling time (mg/L), V is the volume of dye solution (L) and m is the weight of adsorbent (g), it was possible to calculate the adsorption capacity of the powder obtained from MW-treated WPCBs sample. At the end of the adsorption experiment was calculated the experimental equilibrium adsorption capacity (q_e, mg/g).

The experimental data were fitted using the not-linearized equations of various adsorption models, as reported in [1], and the obtained results are reported in **Figure S2**. Fitting was performed using the Solver add-in program of Excel.

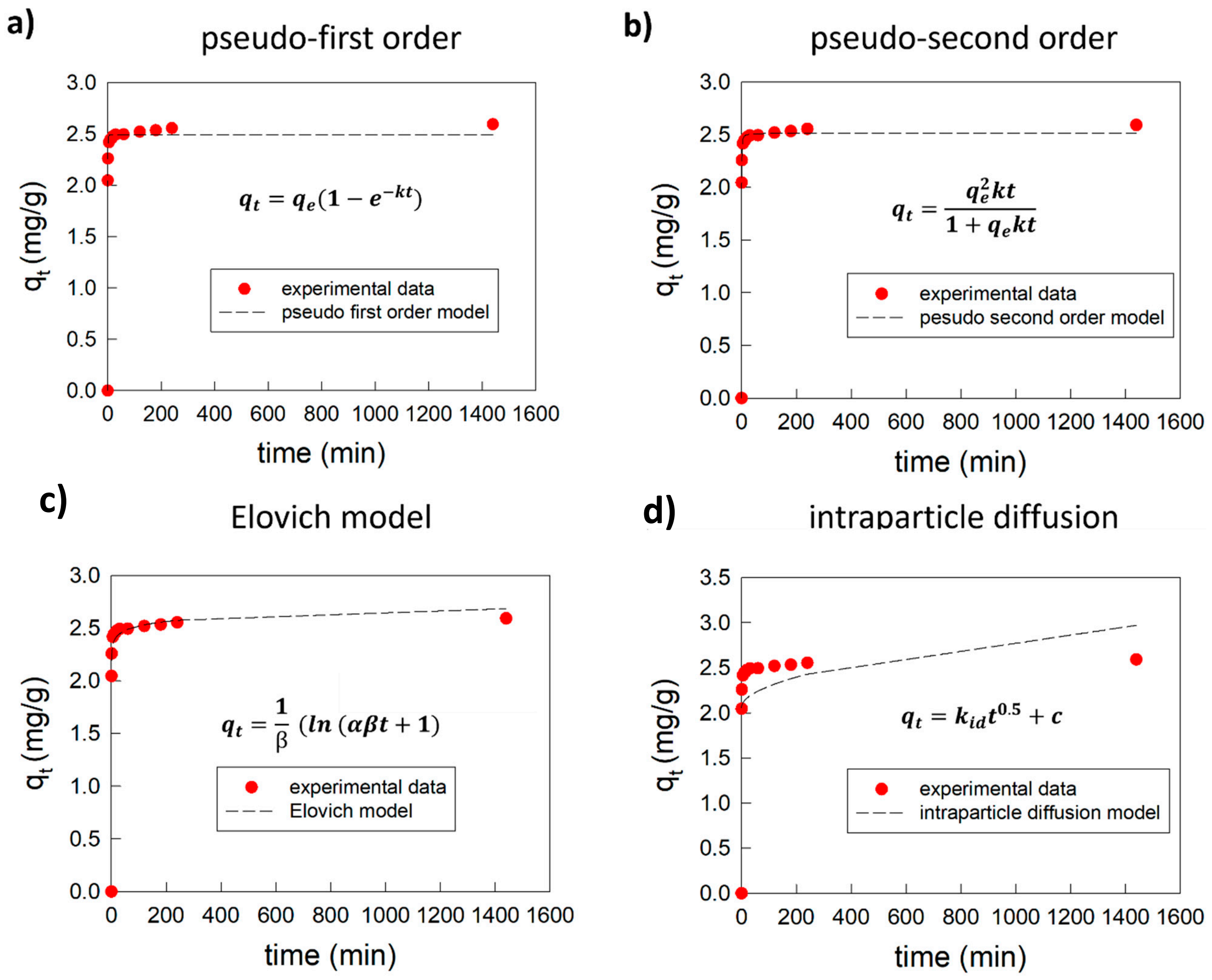

Figure S2. Fitting (black dashed line) of the experimental data (red points) related to the adsorption of MB (10^{-5} M) by the powder derived from MW-treated WPCBs at the dosage of 1.2 mg/L, using different kinetic models, whose equation is reported in the corresponding figure. a) pseudo-first order model; b) pseudo-second order model; c) Elovich model; d) intraparticle diffusion model

For the individuation of the best kinetic model, various statistical parameters (correlation coefficient R^2, adjusted correlation coefficient adj R^2, χ^2, the residual sum of squares error (SSE), the mean sum of squares error (MSE), and the hybrid fractional error function (HYBRID)) were calculated following what reported in [1]. The obtained results are summarized in **Table S2**.

	pseudo first order	pseudo second order	liquid-film diffusion	Elovich	intraparticle diffusion
**R^2**	0.9919	0.9975	0.9919	0.7955	0.1256
**adj R^2**	0.9903	0.9970	0.9903	0.7501	-0.0488
**χ^2**	0.0080	0.0022	0.0080	0.0093	1.1543
**SSE**	0.0465	0.0142	0.0465	0.0506	5.0233
**MSE**	0.0036	0.0011	0.0036	0.0042	0.3864
**HYBRID**	-0.0026	-0.0170	-0.0026	0.0145	8.9695

Table S2. Statistical parameters obtained from the fitting of experimental data related to the adsorption of MB (10^{-5} M) by the powder derived from MW-treated WPCBs at the dosage of 1.2 mg/L, using different kinetic models (not linearized equation).

Table S2 shows that the best results were obtained for the pseudo second order model (PSO). However, in literature has been reported that considering adsorption data very close to the reaching of equilibrium can lead to a wrong attribution of the PSO, instead of the pseudo first order (PFO). For this reason, we repeated the fitting considering adsorption data limited to the first 5 minutes, and the obtained date are reported in **Table S3**. Again, the best results were obtained for the PSO model.

	**pseudo first order**	**pseudo second order**
$\mathbf{R^2}$	0.9951	0.9997
**adj** $\mathbf{R^2}$	0.9852	0.9991
$\boldsymbol{\chi^2}$	0.0046	0.0003
**SSE**	0.0253	0.0016
**MSE**	0.0063	0.0004
**hybrid**	-3.1208	-0.4624

Table S3. Statistical parameters obtained from the fitting of experimental data related to the adsorption of MB (10^{-5} M) by the powder derived from MW-treated WPCBs at the dosage of 1.2 mg/L, considering only the first 5 minutes of the adsorption process. Fitting was performed using the not-linearized equations of the PFO and PSO models.

From the fitting of the experimental data using the PSO model, it was possible to obtain the value of theorical q_e, which corresponded to 2.514 mg/g, showing good accordance with the experimental value (2.593 mg/g).

Section S4. Adsorption of MB at various concentrations

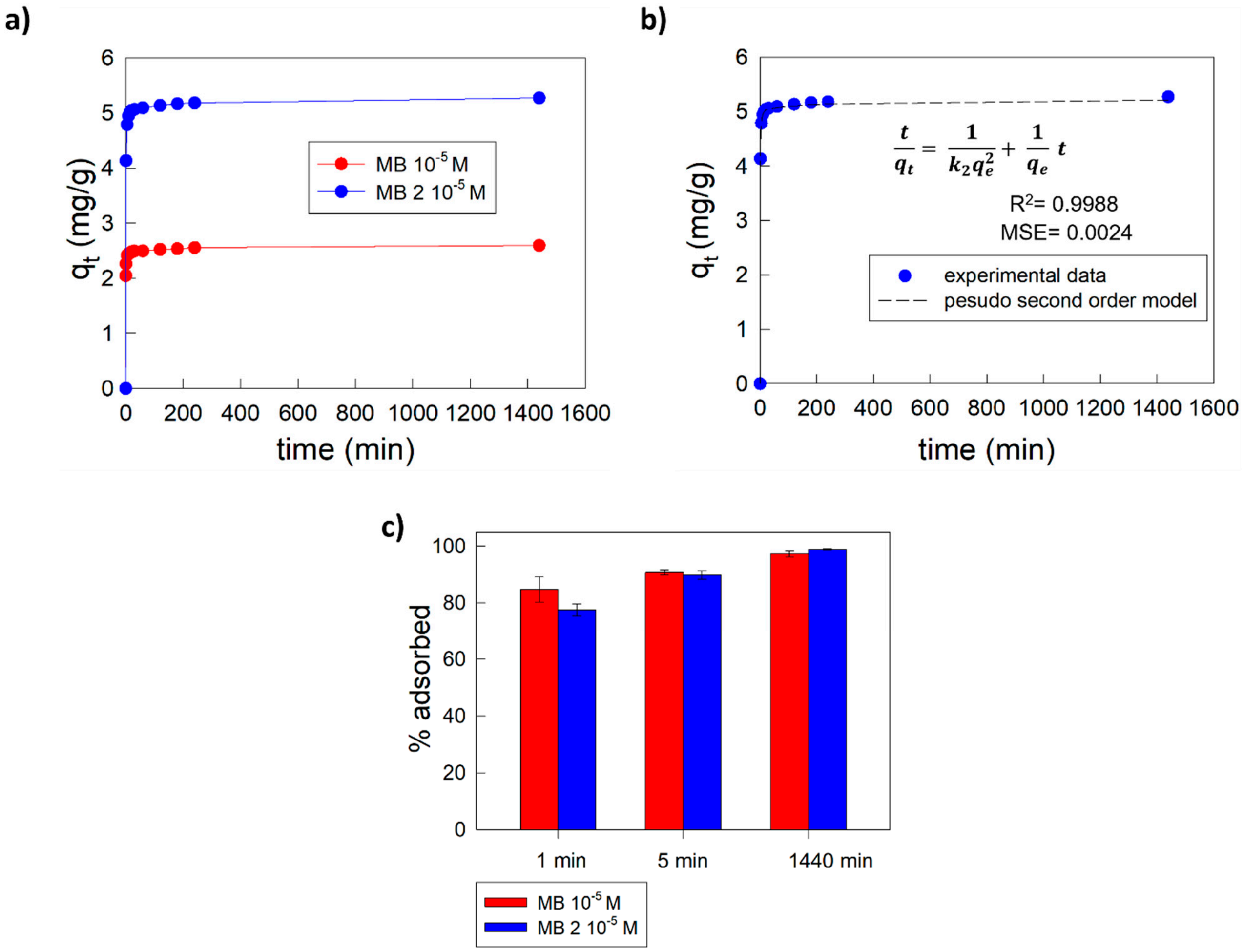

Figure S3. Comparison between the adsorption capacity of MW-treated PCBs powder considering MB at different concentrations, equal to 10^{-5} M and 2 10^{-5} M. a) comparison between the variation of the adsorption capacity as a function of soaking time; b) fitting of the adsorption data related to the MB concentration of 2 10^{-5} M according the not-linearized pseudo-second order model; c) comparison between the % of MB adsorbed at selected time intervals.

Section S5. Fitting of MB adsorption data obtained using different adsorbent materials

The experimental data related to the adsorption of MB 10^{-5}M using different adsorbent powders were fitted using the add-in program Solver in Excel, considering the PFO, PSO, Elovich and intraparticle diffusion kinetic models, similarly to what reported in Section S3 in relation to the powder derived from MW-treated PCBs. In **Figure S4**, the fittings obtained using the best-performing model (selected based on the statistical parameters) are reported. Adsorption of Activated Carbons could be satisfactory modeled by PSO, while the best model for glass powder and untreated PCBs powder was the Elovich model.

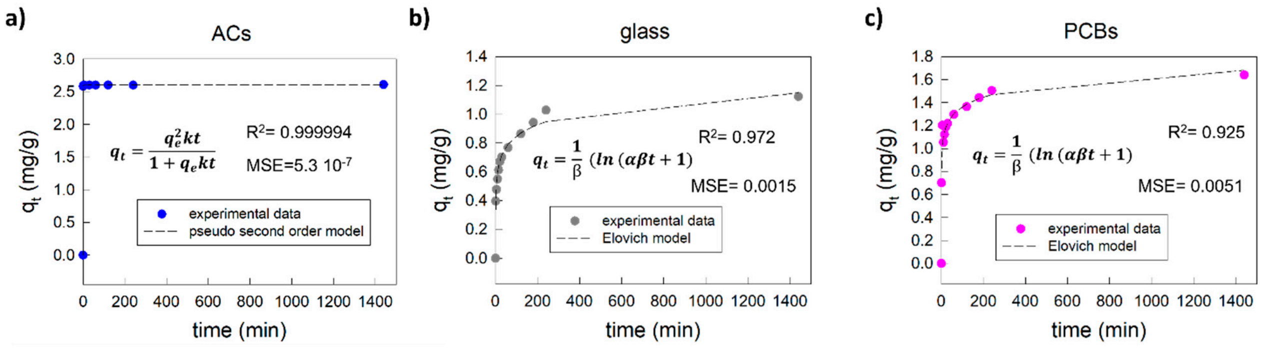

Figure S4. Fitting of the experimental adsorption data of different reference materials for MB 10^{-5} M 10^{-5}M. The equation used for the fitting and related statistical parameters are reported in the figure. a) adsorbent material = activated carbons; b) adsorbent material= glass powder; c) adsorbent material= powder derived from untreated PCBs.

Section S6. Adsorption of MO 10^{-5}M

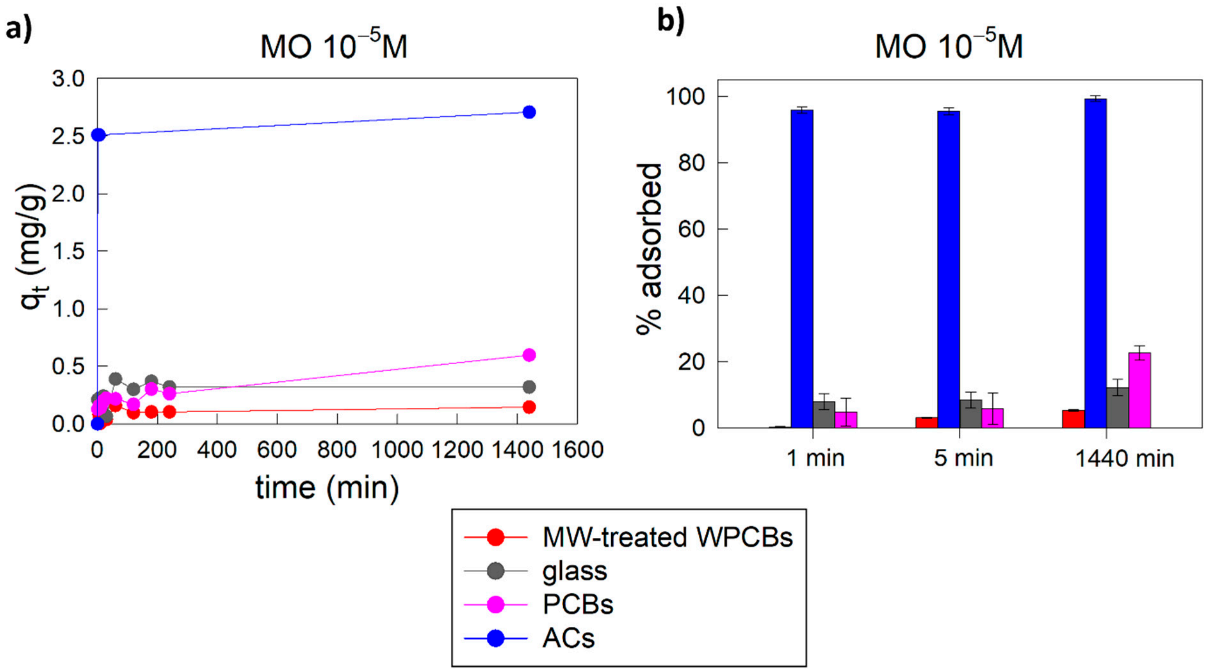

Figure S5. Comparison between the adsorption capacity of MW-treated WPCBs powder and those of reference materials (ACs, glass powder and ground untreated PCBs) during the adsorption of MO (10^{-5}M). a) variation of adsorption capacities as a function of time; b) comparison between the percentage of adsorbed MO 10^{-5} M at selected time intervals. All the experiments were conducted in triplicate, where not visible error bars are included inside dot size.

Section S7. Structural and chemical characterization of Activated Carbons, used as reference material.

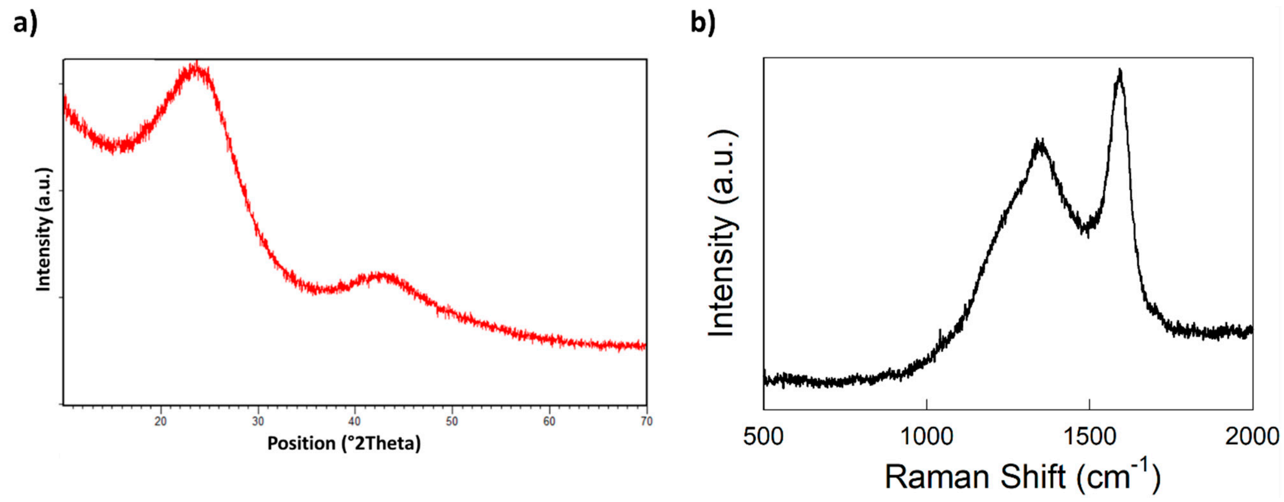

Figure S6. a) X-Ray Diffractogram of ACs and b) Raman spectrum of ACs.

Section S8. Structural and chemical characterization of powder derived from untreated PCBs, used as reference material.

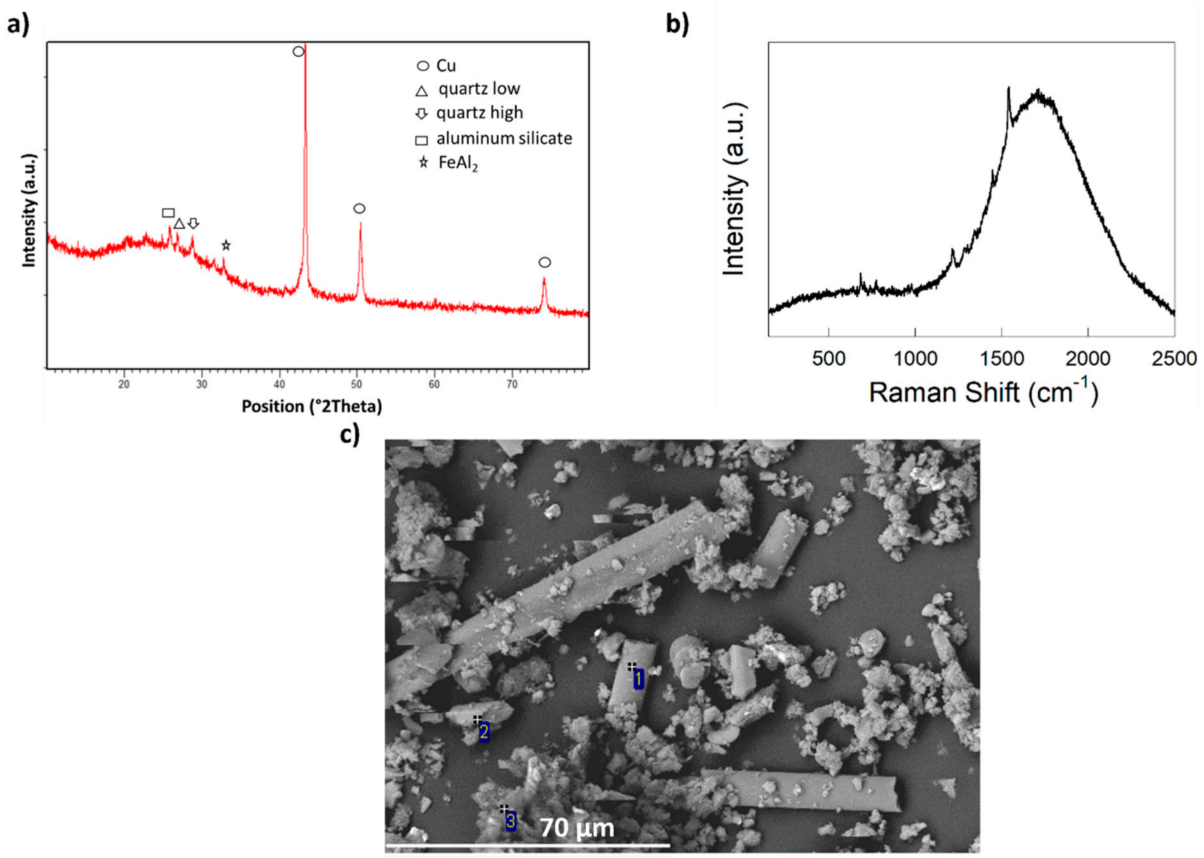

	Spectrum	C	O	F	Al	Si	Cl	Ca	Ti	Cr	Fe	Cu
						wt (%)						
	1	9.84	58.45		5.25	17.53		8.93				
b	2	23.47	51.91		4.38	13.52		6.71				
	3	34.25	48.33		3.22	8.56		4.57				1.08

Figure S7. a) X-Ray Diffractogram of untreated PCBs; b) Raman spectrum of untreated PCBs, c) SEM image and related EDX analysis of untreated PCBs.

References

1. Wang, J.; Guo, X. Adsorption kinetic models: Physical meanings, applications, and solving methods. *J. Hazard. Mater.* **2020**, *390*, 122156, doi:10.1016/j.jhazmat.2020.122156.
